# Randomized phase III study (ADMYRE) of plitidepsin in combination with dexamethasone vs. dexamethasone alone in patients with relapsed/refractory multiple myeloma

**DOI:** 10.1007/s00277-019-03739-2

**Published:** 2019-06-25

**Authors:** Ivan Spicka, Enrique M. Ocio, Heather E. Oakervee, Richard Greil, Raymond H. Banh, Shang-Yi Huang, James M. D’Rozario, Meletios A. Dimopoulos, Sara Martínez, Sonia Extremera, Carmen Kahatt, Vicente Alfaro, Angelo M. Carella, Nathalie Meuleman, Roman Hájek, Argiris Symeonidis, Chang-Ki Min, Paul Cannell, Heinz Ludwig, Pieter Sonneveld, María Victoria Mateos

**Affiliations:** 1 Department of Medicine, Faculty of Medicine, Charles University and General Hospital, Prague, Czech Republic; 2 Department of Hematology, University Hospital Marqués de Valdecilla/IDIVAL, University of Cantabria, Santander, Spain; 3 Department of Haemato-Oncology, St Bartholomew’s Cancer Centre, Barts Health NHS Trust, London, UK; 4 3rd Medical Department, Paracelsus Medical University Salzburg, Salzburg Cancer Research Institute, Cancer Cluster Salzburg, Salzburg, Austria; 5 Department of Clinical Haematology, Mater Health Services, Brisbane, Australia; 6 Department of Medicine, National Taiwan University Hospital, Taipei, Taiwan; 7 Department of Hematology, Canberra Hospital and Health Service, Canberra, Australia; 8 Department of Clinical Therapeutics, Alexandra General Hospital, Athens, Greece; 9 Clinical R&D, Pharma Mar, Colmenar Viejo, Madrid, Spain; 10 Department of Hematology, IRCCS Azienda Ospedaliera Universitaria San Martino, IST Istituto Nazionale per la Ricerca sul Cancro, Genoa, Italy; 11 Department of Hematology, Institut Jules Bordet (ULB), Brussels, Belgium; 12 Department of Hematology-Oncology, Faculty of Medicine, University Hospital Ostrava, Ostrava, Czech Republic; 13 Hematology Division, Department of Internal Medicine, University of Patras Medical School, Patras, Greece; 14 Department of Blood and Marrow Transplantation, Seoul St. Mary’s Hospital, The Catholic University of Korea, Seoul, South Korea; 15 Department of Medicine, Royal Perth Hospital, Perth, Australia; 16 Wilhelminen Cancer Research Institute, Department of Medicine, Center for Oncology, Hematology and Palliative Care, Wilhelminen Hospital, Vienna, Austria; 17 Department of Hematology, Erasmus MC Cancer Institute, Rotterdam, The Netherlands; 18 Department of Hematology, University Hospital of Salamanca/IBSAL, Salamanca, Spain; 19 Departamento de Hematología, Hospital Universitario de Salamanca, Paseo de San Vicente, 58-182, 37007 Salamanca, Spain

**Keywords:** Multiple myeloma, Plitidepsin, Dexamethasone, Relapsed, Refractory

## Abstract

The randomized phase III ADMYRE trial evaluated plitidepsin plus dexamethasone (DXM) versus DXM alone in patients with relapsed/refractory multiple myeloma after at least three but not more than six prior regimens, including at least bortezomib and lenalidomide or thalidomide. Patients were randomly assigned (2:1) to receive plitidepsin 5 mg/m^2^ on D1 and D15 plus DXM 40 mg on D1, D8, D15, and D22 (arm A, *n* = 171) or DXM 40 mg on D1, D8, D15, and D22 (arm B, *n* = 84) q4wk. The primary endpoint was progression-free survival (PFS). Median PFS without disease progression (PD) confirmation (IRC assessment) was 2.6 months (arm A) and 1.7 months (arm B) (HR = 0.650; *p* = 0.0054). Median PFS with PD confirmation (investigator’s assessment) was 3.8 months (arm A) and 1.9 months (arm B) (HR = 0.611; *p* = 0.0040). Median overall survival (OS, intention-to-treat analysis) was 11.6 months (arm A) and 8.9 months (arm B) (HR = 0.797; *p* = 0.1261). OS improvement favoring arm A was found when discounting a crossover effect (37 patients crossed over from arm B to arm A) (two-stage method; HR = 0.622; *p* = 0.0015). The most common grade 3/4 treatment-related adverse events (% of patients arm A/arm B) were fatigue (10.8%/1.2%), myalgia (5.4%/0%), and nausea (3.6%/1.2%), being usually transient and reversible. The safety profile does not overlap with the toxicity observed with other agents used in multiple myeloma. In conclusion, efficacy data, the reassuring safety profile, and the novel mechanism of action of plitidepsin suggest that this combination can be an alternative option in patients with relapsed/refractory multiple myeloma after at least three prior therapy lines.

## Introduction

The prognosis for patients with multiple myeloma (MM) who are refractory to both proteasome inhibitors (PIs) and immunomodulatory drugs (IMiDs) is poor: with further treatment, the median survival is 9 months and 3 months in patients without further treatment [1]. Furthermore, treatment options for MM decrease with each relapse and outcomes with subsequent treatment using standard therapies are characterized by short duration of response and increasing drug resistance [2]. Therefore, there is a need for alternative antimyeloma treatments for patients with advanced illness following refractory/multiply relapsed disease.

Plitidepsin is a cyclic depsipeptide, originally isolated from a Mediterranean marine tunicate, *Aplidium albicans*, which is currently produced by total synthesis. Plitidepsin effects are related to the induction of early oxidative stress, which induces the sustained activation of c-Jun N-terminal kinase (JNK) and p38MAPK and finally apoptosis [3, 4]. The eukaryotic translation elongation factor 1 alpha 2 (eEF1A2), a protein which is overexpressed in MM, has been identified as the primary target for plitidepsin [5]. In vitro studies showed antiproliferative activity against several human MM cell lines [6, 7]. In vivo studies showed an antitumour effect of plitidepsin in xenograft MM models as a single agent or in combination with dexamethasone (DXM) [6]. Plitidepsin plus DXM showed activity in a phase II clinical trial conducted in relapsed/refractory MM patients [8]. Based on the data from the randomized phase III ADMYRE trial, which compared plitidepsin plus DXM with DXM alone, this plitidepsin combination has been recently approved in Australia for the treatment of patients with relapsed/refractory MM who have received at least three prior treatment regimens, including both a PI and an IMiD. Plitidepsin plus DXM has been also approved for its use after two prior lines of therapy in the case of patients refractory and/or intolerant to both a PI and an IMiD. Efficacy and safety results from the ADMYRE trial are presented here.

## Methods

### Patients

Patients were recruited worldwide at 61 investigational sites from 17 countries. The study protocol received protocol assistance by the Committee for Medicinal Products for Human Use (CHMP), was approved by the Independent Local Ethics Committee of each participating center, and was conducted in accordance with the Declaration of Helsinki, Good Clinical Practice guidelines, and local regulations on clinical trials. Signed informed consent was obtained from all patients prior to any study-specific procedure (ClinicalTrials.gov identifier: NCT01102426).

Eligibility criteria included the following: patients ≥ 18 years old with relapsed/refractory MM after at least three, but not more than six, prior therapeutic regimens, including at least bortezomib and lenalidomide or thalidomide; measurable disease; Eastern Cooperative Oncology Group performance status (ECOG PS) ≤ 2; life expectancy ≥ 3 months; and adequate major organ function. Patients were excluded if they had the following: a concomitant unstable or serious medical condition (e.g., myocardial infarction, angina, congestive heart failure, severe dyspnea or oxygen requirement, active uncontrolled infection, immune deficiency, or myopathy), grade > 2 peripheral neuropathy, myelodysplasia and/or post-chemotherapy aplasia, or mood disturbance associated with previous steroid-based therapy.

### Treatment

In 2009, at the time of designing the ADMYRE trial, no standard treatment existed for the intended population (MM patients relapsed/refractory to all standard available therapy) that could be considered a gold standard comparator, and treatment options were limited. DXM was an active compound widely used as a single agent as well as part of combination regimens for treatment of MM patients in different settings. For that reason, the European Society of Medical Oncology recommendations included DXM added to these agents as treatment for relapsed/refractory MM [9]. On this basis, the choice of the control arm (low-dose DXM) was agreed with the health authorities (European Medicines Agency and Food and Drug Administration of the USA).

Patients were stratified according to their ECOG PS score (0 and 1 vs. 2) and Durie-Salmon stage (I/II vs. III) and were randomly assigned (with a 2:1 ratio) to receive plitidepsin 5 mg/m^2^ on D1 and D15 intravenously (i.v.) over 3 h plus DXM 40 mg orally on D1, D8, D15, and D22 (arm A) or DXM 40 mg orally on D1, D8, D15, and D22 (arm B), every 4 weeks (q4wk). Patients in the control arm (DXM alone, arm B) with documented disease progression after a minimum of 8 weeks from randomization could crossover to the combination arm (arm A).

### Efficacy assessment

The primary efficacy endpoint was progression-free survival (PFS), which was assessed according to an Independent Review Committee (IRC) per the International Myeloma Working Group (IMWG) criteria current at the time of study protocol design (i.e., without requiring PD confirmation for assigning a PFS event) [2]. Furthermore, a preplanned sensitivity analysis of PFS by an investigator’s assessment was performed following the revised IMWG criteria (i.e., with PD confirmation required for assigning a PFS event) [10]. Secondary efficacy endpoints were objective response rate (ORR; ≥ partial response) according to the IMWG criteria, duration of response, and overall survival (OS).

### Safety assessment

Safety was evaluated in all patients who received at least one dose of the study treatment by assessment of adverse events (AEs), clinical laboratory test results, physical examinations, and vital signs. AEs were recorded and coded with the Medical Dictionary for Regulatory Activities (MedDRA) v.16.0. AEs and laboratory values were graded according to the National Cancer Institute-Common Toxicity Criteria for Adverse Events (NCI-CTCAE) v.4. All patients were followed until recovery from any treatment-related AE. Follow-up was longer in arm A partly because it included patients that had crossed over from arm B.

### Statistical methods

The number of randomized patients required was calculated on the basis of PFS estimates from the previous phase II study in MM [8]. Approximately 210 progression or death events were required to reject the equality of hazard rates (HR) between both treatment arms, assuming a HR of 0.625 in favor of the combination arm (90% power, 1-sided 2.5% significance level). A futility analysis was done once 40 patients in arm A were evaluable for response. ORR including minor response (MR) was 37.8% (planned threshold was 30%), and therefore patient accrual was resumed. The final PFS analysis was done with data obtained in November 2015.

The unstratified log-rank test was used to compare PFS. Cox regression was used to calculate the risk reduction in PFS. Binomial estimates with exact 95% confidence intervals (CIs) were calculated for the analysis of ORR. Fisher’s exact test was used to compare ORR.

**Fig. 1:**
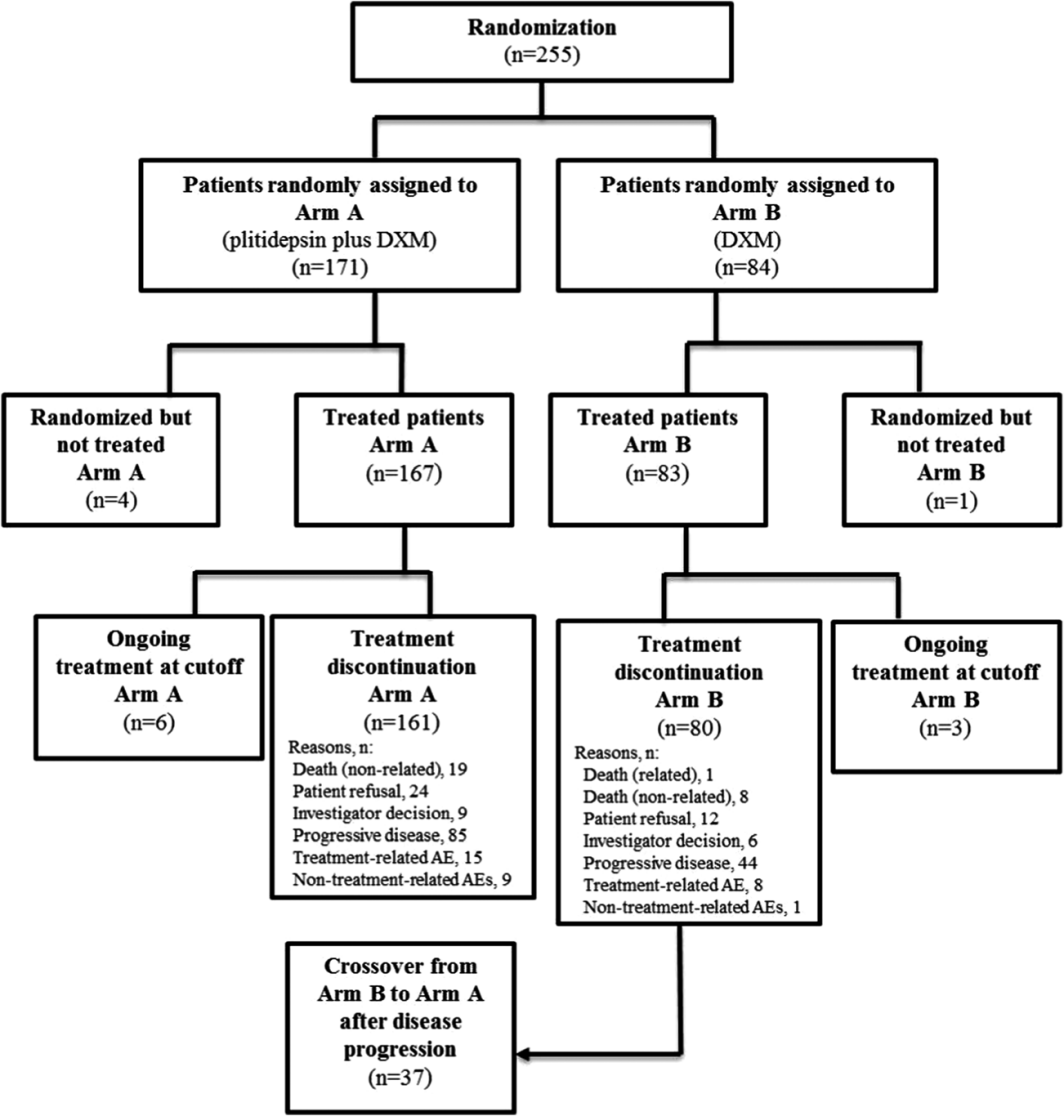
Study flow chart. AE, adverse event; DXM, dexamethasone

The study was powered for the evaluation of the main endpoint, PFS, and for ascertaining if a trend in OS is observed in favor of the experimental arm. The final OS analysis was done according to the Kaplan-Meier method 2 years after the last patient inclusion (May 2017). Nevertheless, the potential to detect statistically significant differences in OS was hampered by crossover: 44.0% of patients from arm B crossed over to arm A (Fig. 1). Then, a post hoc sensitivity analysis based on the two-stage method proposed by Latimer et al. [11], which was previously reported when evaluating OS in another MM trial [12], was performed.

## Results

### Patient characteristics

A total of 255 patients were randomized (2:1) between June 2010 and May 2015: 171 in arm A and 84 in arm B. Of these, 167 patients were treated in arm A and 83 in arm B (Fig. 1). Baseline characteristics were well balanced between treatment arms (Table 1). The median number of lines of previous therapy was 4 in both treatment arms. In both arms, around 74% of patients had refractory or relapsed and refractory disease to the last line (38–39% of them relapsed and refractory to lenalidomide/thalidomide and bortezomib).

**Table 1:** Patient baseline characteristics

		Arm A (plitidepsin plus DXM) (*n* = 171)		Arm B (DXM) (*n* = 84)	
		*n*	%	*n*	%
Gender	Female	74	43.3	49	58.3
	Male	97	56.7	35	41.7
Age (years)	Median (range)	64 (36–85)		65 (42–85)	
ECOG PS at baseline	0	68	39.8	31	36.9
	1	74	43.3	42	50.0
	≥ 2	29^a^	16.9	11	13.1
MM type at diagnosis	Non-secretory	6	3.5	1	1.2
	Secretory	165	96.5	83	98.8
MM type at baseline^b^	Non-secretory	17	10.1	2	2.4
	Secretory	151	89.9	80	97.6
Durie-Salmon stage at diagnosis	I	21	12.4	11	13.1
	II	48	28.1	21	25.0
	III	101	59.1	51	60.7
Cytogenetic risk group at baseline^c^	Standard risk	47	27.5	22	26.2
	High risk	45	26.3	20	23.8
	Not available	79	46.2	42	50.0
Beta-2 microglobulin (mg/L)	Median (range)	4.1 (0.0–27.3)		4.2 (0.2–65.0)	
Hemoglobin (g/dL)	Median (range)	10.4 (7.0–14.6)		10.1 (7.4–14.6)	
Platelets (10^9^/L)	Median (range)	140 (11.0–517.0)		154.0 (24.0–452.0)	
Neutrophils (10^9^/L)	Median (range)	2.2 (0.5–7.6)		2.3 (0.5–10.3)	
CL_CR_ (mL/min)	Median (range)	72.9 (21.9–252.2)		69.4 (23.0–137.0)	
% plasma cells	Median (range)	40.0 (0.0–100.0)		33.3 (0.0–100.0)	
Disease status					
Relapsed and refractory to lenalidomide/thalidomide and bortezomib		65	38.0	33	39.3
Relapsed and refractory to lenalidomide/thalidomide but not to bortezomib		39	22.8	24	28.6
Relapsed and refractory to bortezomib but not to lenalidomide/thalidomide		20	11.7	12	14.3
Other than above		47	27.5	15	17.9
Disease status with respect to the last prior therapy^d,e^	Relapsed	34	19.9	15	17.9
	Total refractory	126	73.7	62	73.8
	Refractory	71	41.5	39	46.4
	Relapsed and refractory	55	32.2	23	27.4
	Unknown	11	6.4	7	8.3
Prior stem cell transplantation	Number of SCT				
	0	56	32.7	29	34.5
	1	82	48.0	39	46.4
	≥ **2**	33	19.3	16	19.0
	Type				
	Allogenic	6	5.2	5	9.1
	Autologous	115	100.0	54	98.2

Data shown are randomized patients; *n* (%) or median (range)

^a^One patient in arm A had ECOG PS = 1 at randomization but ECOG PS = 3 before starting the study treatment

^b^Five patients had no MM type defined at baseline: three in arm A and two in arm B. No marked differences were found between the two treatment arms, at both diagnosis and baseline

^c^High risk: patients with translocations such as t(4;14), t(14;16), t(14;20), del 17, and del 13 or single alterations such as + 1q or + 1p. Standard risk: patients with translocations such as t(11;14), t(6;14) or other, as well as patients with single alterations of trisomies 3, 5, 6, 9, 11, 15, 19, or 21

^d^*Relapsed* MM was defined as previously treated myeloma that progressed and required the initiation of salvage therapy but did not meet the criteria for either “refractory” or “relapsed and refractory” myeloma categories

*Total refractory* MM included two categories of refractory myeloma:

*•Refractory* MM was defined as disease that was non-responsive in patients who had never achieved a MR or better, with any therapy. It included patients who never achieved MR or better, in whom there was no significant change in monoclonal protein (M-protein) and no evidence of clinical progression, as well as primary refractory PD where patients met criteria for true PD

*•Relapsed and refractory* MM was defined as disease that was non-responsive while on salvage therapy or progressed within 60 days of the last therapy in patients who had achieved MR or better at some point before progressing

^e^The most common previous agents were bortezomib (98.4% of patients), lenalidomide (97.6%), DXM (97.6%), melphalan (87.8%), cyclophosphamide (74.5%), thalidomide (65.1%), doxorubicin (47.1%), vincristine (32.5%), and prednisone (23.9%). Other prior, more novel anti-MM agents included pomalidomide (13.3%), carfilzomib (3.9%), vorinostat (2.7%), elotuzumab (2.0%), and panobinostat (1.2%)

*CL*_*CR*_ creatinine clearance, *DXM* dexamethasone, *ECOG* Eastern Cooperative Oncology Group, *MM* multiple myeloma, *MR* minor response, *PD* disease progression, *PS* performance status

### Efficacy

**Fig. 2:**
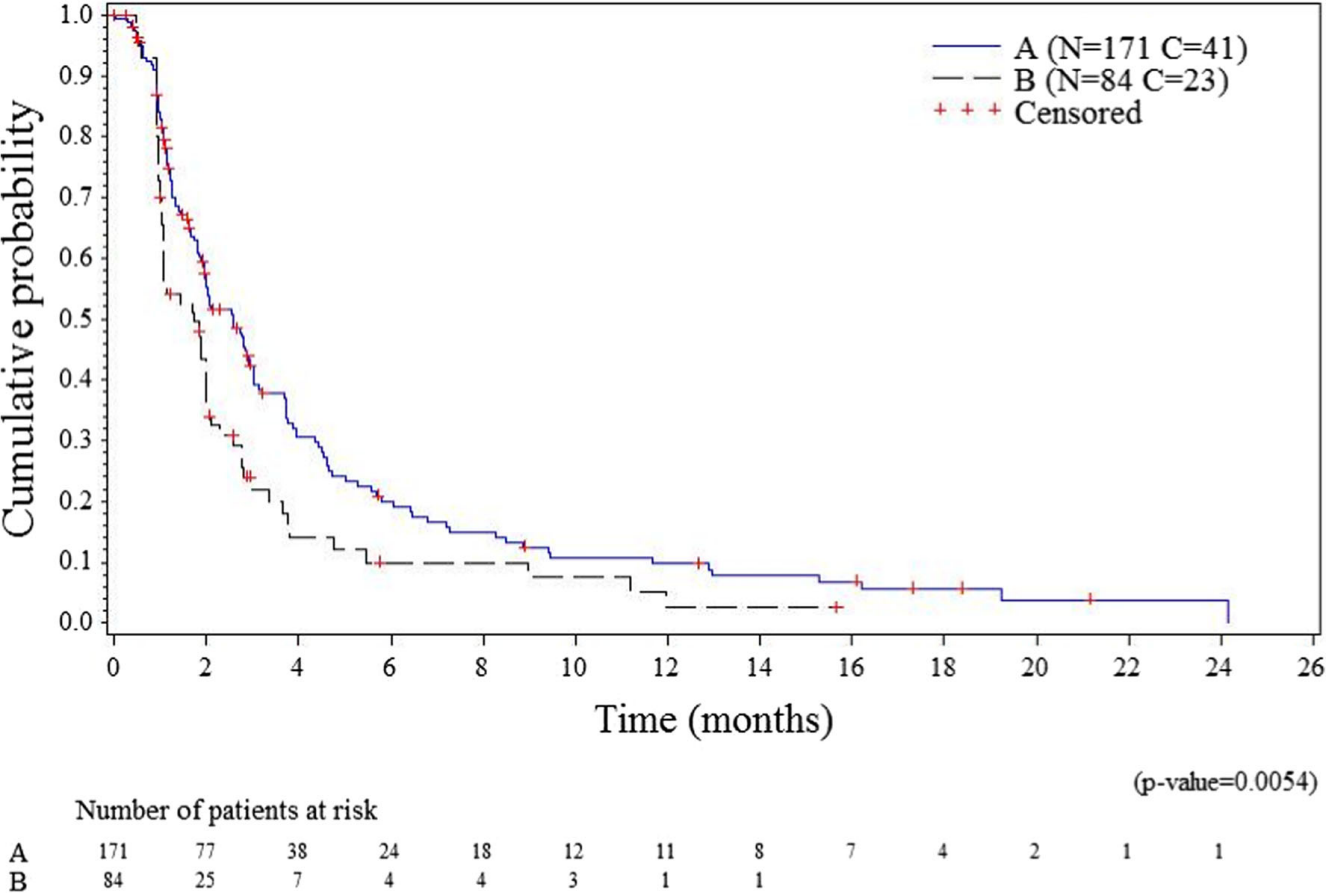
Kaplan-Meier curve for progression-free survival by the Independent Review Committee. A, arm A (plitidepsin plus DXM); B, arm B (DXM); C, censored; DXM; dexamethasone; N, number of patients

The primary efficacy analysis, with blinded IRC assessment of all randomized patients done without PD confirmation, showed statistically significant longer PFS for patients treated with plitidepsin plus DXM. Median PFS was 2.6 months (95% CI, 1.9–3.0 months) in arm A (plitidepsin plus DXM) and 1.7 months (95% CI, 1.1–2.0 months) in arm B (DXM) (log-rank *p* = 0.0054) (Fig. 2). The relative risk of progression or death was reduced by 35.0% in patients treated with plitidepsin plus DXM (HR = 0.650; 95% CI 0.477–0.885, *p* = 0.0062).

PFS analysis requiring PD confirmation by an investigator’s assessment showed a median PFS of 3.8 months (95% CI, 2.9–5.6 months) in arm A (plitidepsin plus DXM) and 1.9 months (95% CI, 1.1–2.7 months) in arm B (DXM), with a relative risk of progression or death reduced by 38.9% in patients treated with plitidepsin plus DXM (HR = 0.611; *p* = 0.0048).

ORR according to the IRC in evaluable patients was 13.8% (95% CI, 8.3–21.2%) in arm A (plitidepsin plus DXM; *n* = 123), which included two very good partial responses and 15 partial responses (median duration of response was 12.0 months), and 1.7% (95% CI, 0.04–9.1%) in arm B (DXM; *n* = 59) (one patient with partial response; duration of 1.8 months) (*p* < 0.0080).

The clinical benefit rate, defined as patients with response (including MR) or stable disease (SD), was 48.0% (95% CI, 40.3–55.7%) in arm A (plitidepsin plus DXM) and 28.6% (95% CI, 19.2–39.5%) in arm B (DXM) (*p* < 0.0044).

**Fig. 3:**
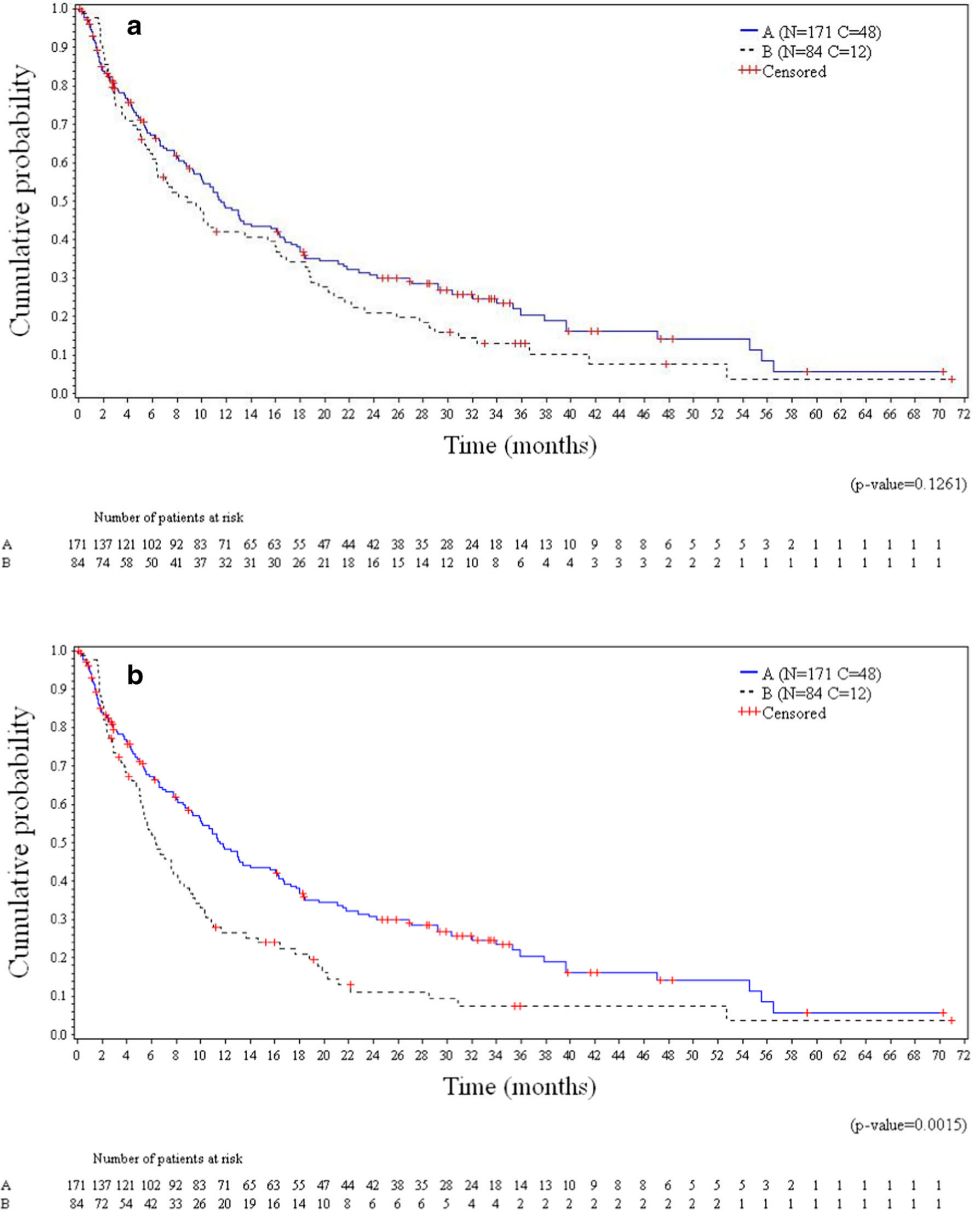
Kaplan-Meier curves for overall survival in all randomized patients (**a**) and all randomized patients (two-stage method) (**b**). A, arm A (plitidepsin plus DXM); B, arm B (DXM); C, censored; DXM, dexamethasone; N, number of patients

**Fig. 4:**
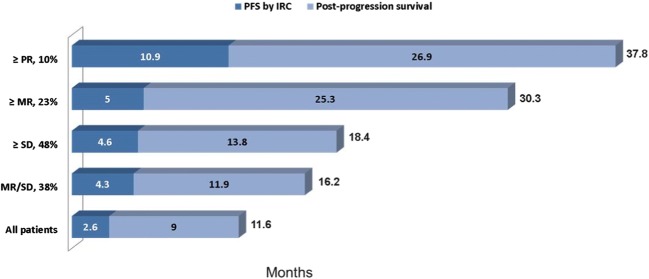
Progression-free survival, post-progression survival, and overall survival according to the response observed. IRC, Independent Review Committee; MR, minor response; PFS, progression-free survival; PR, partial response; SD, stable disease

The final intention-to-treat OS analysis was based on 195 death events (76.5% of the 255 randomized patients). Median OS was 11.6 months (95% CI, 9.2–16.1 months) in arm A (plitidepsin plus DXM) and 8.9 months (95% CI, 6.0–15.4 months) in arm B (DXM) (log-rank *p* = 0.1261) (Fig. 3a). Despite crossover, relative risk of death was reduced by 20.3% in patients treated with plitidepsin plus DXM (HR = 0.797; *p* = 0.1273). Median OS for patients with response or clinical benefit in arm A (plitidepsin plus DXM) was 37.8 months and 30.3 months, respectively (Fig. 4).

A two-stage OS analysis, which mitigates the effect of crossover, showed a statistically significant difference in favor of arm A (plitidepsin plus DXM). Median OS remained as 11.6 months (95% CI, 9.2–16.1 months) in arm A (plitidepsin plus DXM) and was estimated as 6.4 months (95% CI, 5.1–8.3 months) in arm B (DXM) (log-rank *p* = 0.0015) (Fig. 3b). Relative risk of death was reduced by 37.8% in patients treated with plitidepsin plus DXM (HR = 0.622; *p* = 0.0016).

### Safety

All treated patients were evaluable for safety. The median (range) of cycles received was 3 (1–33) in arm A (plitidepsin plus DXM; total cycles = 842) and 2 (1–21) in arm B (DXM; total cycles = 251). Median time on treatment was 12.3 weeks (1.3–137.1 weeks) in arm A and 8.3 weeks (1.4–85.3 weeks) in arm B. Fifteen patients (9.0%) discontinued treatment because of treatment-related AEs in arm A and eight patients (9.6%) in arm B.

In arm A (plitidepsin plus DXM), the most common AEs (all grades) related to the study treatment (or with unknown causality) were nausea (37.1% of patients), fatigue (36.5%), vomiting (16.8%), diarrhea (14.4%), myalgia (14.4%), peripheral edema (12.0%), decreased appetite (12.6%), and muscular weakness (9.6%). The most common grade 3/4 treatment-related (or with unknown causality) AEs were fatigue (10.8%), myalgia (5.4%), muscular weakness (3.6%), and nausea (3.6%) (Table 2). Other grade 3/4 AEs of specific interest were as follows: creatine phosphokinase (CPK) increase (20.0%), alanine aminotransferase (ALT) increase (14.5%), peripheral sensory neuropathy (0.6%), and infection/pneumonia (2.4%). One patient died following a treatment-related AE (grade 4 myopathy) after having received one cycle (*n* = 1/167 patients; 0.6%).

**Table 2:** The most common laboratory abnormalities (regardless of relationship) and treatment-related adverse events (≥ 10% of patients)

	Arm A (plitidepsin plus DXM) (*n* = 167)						Arm B (DXM) (*n* = 83)^a^					
	NCI-CTCAE grade						NCI-CTCAE grade					
	All		3		4		All		3		4	
	*n*	%	*n*	%	*n*	%	*n*	%	*n*	%	*n*	%
Hematological abnormalities (regardless of relationship)^b^												
Anemia	157	98.1	48	30.0	2	1.3	77	97.5	26	32.9	2	2.5
Lymphopenia	110	68.8	32	20.0	5	3.1	54	69.2	11	14.1	2	2.6
Thrombocytopenia	95	59.4	21	13.1	14	8.8	53	67.1	9	11.4	13	16.5
Leukopenia	84	52.5	14	8.8	2	1.3	37	46.8	2	2.5	.	.
Neutropenia	76	47.5	22	13.8	3	1.9	33	42.3	3	3.8	1	1.3
Biochemical abnormalities (regardless of relationship)^b^												
Increased ALT	135	84.9	20	12.6	3	1.9	16	20.3	.	.	.	.
Increased creatinine	132	82.5	2	1.3	1	0.6	70	88.6	2	2.5	1	1.3
Increased AST	103	66.0	13	8.3	1	0.6	19	24.4	.	.	.	.
Increased CPK	69	44.5	13	8.4	18	11.6	3	4.3	.	.	.	.
Increased ALP	49	31.0	2	1.3	1	0.6	10	13.0	.	.	.	.
Increased bilirubin	18	11.3	3	1.9	.	.	7	8.9	.	.	.	.
Adverse events (treatment-related or with unknown relationship)												
Nausea	62	37.1	6	3.6	.	.	9	10.8	1	1.2	.	.
Fatigue	61	36.5	17	10.2	1	0.6	7	8.4	1	1.2	.	.
Myalgia	24	14.4	7	4.2	2	1.2	2	2.4	.	.	.	.
Vomiting	28	16.8	3	1.8	.	.	2	2.4	1	1.2	.	.
Diarrhea	24	14.4	2	1.2	.	.	2	2.4	.	.	.	.
Peripheral edema	20	12.0	2	1.2	.	.	2	2.4	.	.	.	.
Decreased appetite	21	12.6	1	0.6	.	.	2	2.4	.	.	.	.

Data shown are *n* (%) of patients

Ordered by frequency

^a^Events occurring after crossover are excluded from this table

^b^Percentages based on total patients with laboratory data available

*ALP* alkaline phosphatase, *ALT* alanine aminotransferase, *AST* aspartate aminotransferase, *CPK* creatine phosphokinase, *DXM* dexamethasone, *NCI-CTCAE* National Cancer Institute-Common Terminology Criteria for Adverse Events

In arm B (DXM), the most common AEs (all grades) related to the study treatment (or with unknown causality) were nausea (10.8%), fatigue (8.4%), and insomnia (9.6%). All grade 3/4 AEs occurred in one (1.2% of patients) or two patients (2.4%) each (Table 2). One patient died following a treatment-related AE (grade 4 respiratory tract infection) after having received two cycles (*n* = 1/83 patients; 1.2%).

## Discussion

The ADMYRE study met its primary endpoint, demonstrating a 35% reduction in the relative risk of progression or death for the combination of plitidepsin plus DXM compared with DXM alone in relapsed/refractory MM patients pretreated with at least three regimens, including bortezomib and either lenalidomide or thalidomide. Short PFS values reported in the primary PFS analysis (2.6 months in arm A; 1.7 months in arm B) can be explained by the conservative adjudication of PD time points, which were calculated according to the IMWG criteria version current when the study was planned and designed [2], but also to the advanced stage of the heavily pretreated population, who generally had exhausted most available therapies. A preplanned sensitivity analysis of PFS in line with updated IMWG criteria [10], which require confirmation of PD in two consecutive assessments, showed a longer PFS value for the combination (3.8 months), with the difference between treatments reaching almost 2 months.

The reduction in the relative risk of death observed with mature survival data (23.5% of patients censored) was 20.3% despite the substantial crossover rate (44.0% of patients). The finding of a similar outcome in survival, a secondary but robust endpoint, when discounting the effect of crossover in the two-stage method (statistically significant 37.8% reduction in the risk of death relative to the control arm) supports the benefit demonstrated in PFS, the primary study endpoint (statistically significant 35.0% reduction in the relative risk of progression or death). Survival results for patients with response (median OS of 37.8 months, with a median duration of response of 12.0 months) or clinical benefit (median OS of 30.3 months) are of interest in the setting of a pretreated MM patient population (expected median survival is about 9 months).

Several drugs (carfilzomib, ixazomib, pomalidomide, daratumumab, elotuzumab, and panobinostat) have been more recently approved for the treatment of patients in whom the use of bortezomib and lenalidomide has been exhausted. Beyond these, there are few options for salvage treatment, which are limited to re-challenge with a previously used treatment (alone or in combination with corticosteroids or other novel agents), use of older drugs (e.g., thalidomide, melphalan, vincristine, doxorubicin, etoposide, bendamustine, and carmustine), or enrolment in a clinical trial [1].

Carfilzomib (a new irreversible PI) was evaluated in a phase II trial in patients pretreated with five lines of therapy (95% of whom had disease refractory to the last therapy) [13]. Median PFS was 3.7 months, and median OS in patients refractory to both bortezomib and lenalidomide was 15.6 months. The data from the ADMYRE study was obtained in a population less heavily pretreated (four prior lines; 74% of patients with refractory disease). Nevertheless, a median PFS of 3.8 months (updated IMWG criteria) [10] and a median OS of 11.6 months observed with plitidepsin plus DXM confirm a level of activity close to that observed for carfilzomib.

Daratumumab, a human monoclonal IgG1 antibody that binds to the CD38 myelomatous cell surface antigen, was evaluated in a phase II study (SIRIUS) in patients who had received at least three prior lines of therapy or were refractory to the most recent PI and IMiD combination [14]. The median duration of response was 7.4 months and median PFS was 3.7 months. In the ADMYRE study, conducted in a heavily pretreated and largely refractory population, the median duration of response was longer (12.0 months) in patients achieving VGPR or PR, and median PFS (updated IMWG criteria) was similar (3.8 months).

Pomalidomide plus low-dose DXM was evaluated in a randomized phase III study (MM-003) in MM patients pretreated with a median of five previous regimens (82% of them with refractory disease status) [15]. The median PFS (4.0 months) and median OS (12.7 months) were quite similar to the ADMYRE results.

Plitidepsin plus DXM showed a low incidence of toxicities that are common with available agents used in the treatment of relapsed/refractory MM, such as venous thromboembolism, neurotoxicity, neutropenia and associated infections, thrombocytopenia and associated bleeding, or cardiac events [13–19], which represents a favorable safety profile in a disease setting of heavily pretreated patients. Most of the toxicities observed with plitidepsin plus DXM were transient, non-cumulative laboratory abnormalities that usually occur in the first two cycles of treatment and are controlled by dose adjustment (cycle delay, dose omission, and in ultimate instance, dose reduction). eEF1A2 is overexpressed in MM [20] and has been identified as the primary intracellular target of plitidepsin [5, 21]. eEF1A2 is responsible for the enzymatic delivery of aminoacyl-tRNAs to the ribosome, but also has pro-oncogenic activities including regulation of oxidative stress [22], inhibition of apoptosis [23], or control of unfolded protein degradation by the proteasome [24]. All recently introduced new myeloma drugs have mechanisms of activity not targeting eEF1A2; this fact, together with the favorable safety profile and the lack of overlapping toxicities with commonly used agents, places plitidepsin as an alternative option for designing combinations or even for its administration after relapse in patients treated with immunotherapy.

In conclusion, the combination of plitidepsin and DXM has shown antimyeloma activity compared with DXM alone, introduces a new agent with a novel mechanism of action into the MM therapeutic armamentarium, has an acceptable safety profile different from that of PIs, IMiDs, or histone deacetylase inhibitors, and could thus be considered an alternative treatment option for patients with relapsed/refractory MM.
